# Freight Traffic Impacts and Logistics Inefficiencies in India: Policy Interventions and Solution Concepts for Sustainable City Logistics

**DOI:** 10.1007/s40890-022-00161-8

**Published:** 2022-07-07

**Authors:** Prasanta K. Sahu, Agnivesh Pani, Georgina Santos

**Affiliations:** 1 Department of Civil Engineering, BITS Pilani, Hyderabad, 500078 India; 2 Department of Civil Engineering, Indian Institute of Technology (BHU), Varanasi, India; 3 School of Geography and Planning, Cardiff University, Cardiff, UK

**Keywords:** Freight policies, Urban freight, Sustainability, Logistics performance, Trucking, India

## Abstract

Freight traffic fulfils not only the business needs of a region to move goods between producers, manufacturers, and end consumers, but also creates a host of unintended environmental, social, and economic impacts. Despite its importance, freight traffic impacts and associated logistic inefficiencies are largely overlooked in the urban transport discussions in developing economies like India. This paper addresses this research gap by outlining the research progress related to freight transport in India and discusses the key problems related to freight system performance. The published literature in the last three decades (1990–2020), policy briefs and institutional reports are explored to summarize key findings and uncover thematic linkages. We categorize the inefficiencies in the freight system into four aspects: (i) long-haul trucking, (ii) last-mile logistics, (iii) freight distribution (inventory level), and (iv) policies and regulations. Apart from identifying the limitations in policy discourse, this paper also explores the possible solution concepts to improve efficiency in freight transport and mitigate the unintended negative externalities in urban areas. The overall conclusion is that increasing and improving infrastructure and equipment, technology and operations, and policy and regulations will go some way towards making freight more efficient in India and reducing congestion and emissions of air pollutants and GHG. The present paper can be expected to promote further freight research and effective policy instrument design in India.

## Introduction

Currently, more than a third of global transport energy consumption (39%) is generated by freight movements [1]; trucking is responsible for 23%, followed by marine vessels, which are responsible for 12%, and rail and pipelines, which are responsible for 4%. It is therefore an imperative research need to investigate how to ensure that freight activities fulfil their role in economic transaction of goods, while mitigating the associated negative externalities. It is also critically important to understand why, how and where freight activity takes place and what kind of infrastructure and policies need to be provided to respond effectively to the growing logistical requirements of businesses and households [2–4]. The practical requirements to improve the logistics competency and operational efficiency of freight transport have been acting as strong catalysts to stimulate a number of studies towards understanding freight activity at both national and local scales. However, a data-driven summary of the freight system performance in India, expanding on the logistics inefficiencies and negative externalities of freight traffic, are evident gaps in the literature. This discernible research need triggered the present comprehensive review. This review specifically outlines the progress that has been made in freight research, along with possible future research directions and policy guidelines.

The objectives of this review paper are therefore threefold: (1) to investigate the various aspects of freight system performance, logistics inefficiencies, and freight traffic negative externalities in India; (2) to discuss the potential solution concepts and prepare a research agenda for future research on sustainable city logistics in India; and (3) to develop insights for policy and practice based on the empirical evidence in the literature and the emerging trends in the logistics market. These objectives are motivated by the lack of practice- and policy-based discussions on improving freight mobility in India and enhancing the ease in moving goods across cities and states in a geographically diverse country like India. Reviewing the inefficiencies and externalities of freight in India will help to provide solution concepts and mitigation strategies to improve the freight system performance. This paper, therefore, addresses the need for a comprehensive review focusing on Indian freight studies and aims to draw inferences from the existing literature and provide guidance for future research in India. The review findings are expected to promote freight research and effective policy instrument design to meet the growing needs to reduce the overall logistics cost for moving goods.

## Method Adopted for the Review and Data Collection

### Research Questions

The primary aim of this paper is to present a comprehensive review of the freight traffic impacts and logistics inefficiencies in India, which is an area of significant practical and research interest in the context of coordinated global efforts to reduce transport emissions. The following are the specific research questions explored in this review:

1. What is the extent of literature relevant to freight transport planning in India and what is the emerging trend?
2. Are there any deficiencies of freight performance in India relative to global benchmarks?
3. What are the logistics inefficiencies in India and what are the underlying reasons contributing to them?
4. What are the environmentally negative externalities of freight movements and how can they be quantified in India?
5. What are the potential solution concepts that can be derived from the literature for addressing the issues related to freight system performance, logistics inefficiencies, and negative externalities of freight movements in India?

**Fig. 1:**
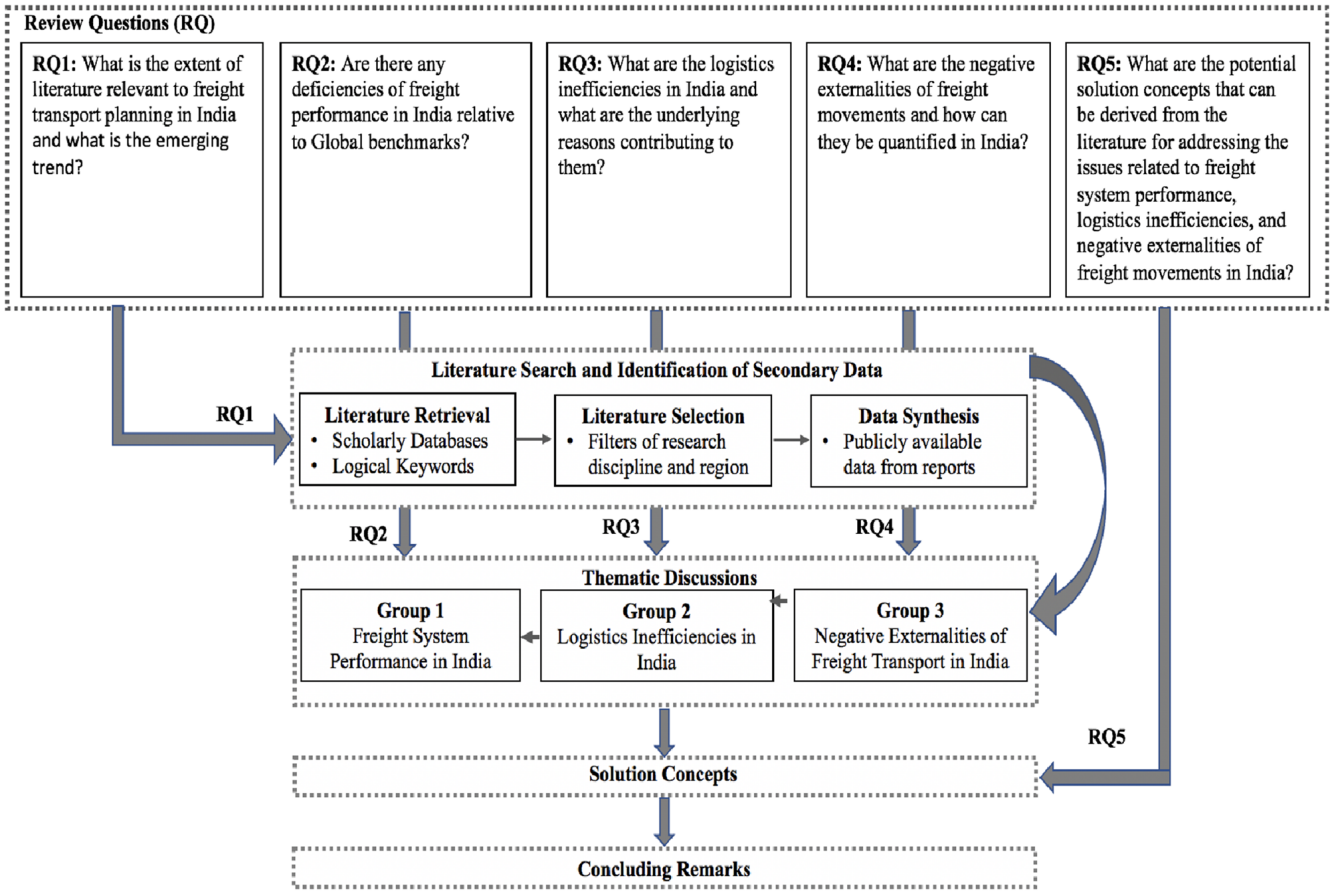
Overview of the review questions, method, and discussion structure

An overview of the review questions, methodology and the discussion structure adopted in this paper is presented in Fig. 1. As can be seen, the first research question (RQ1) is designed to map the extent of literature related to freight transport planning in India through a review of published literature, policy documents, and reports. The second research question (RQ2) follows from the previous question as it is to analyse and discuss the freight system performance in India based on the papers identified and screened as a part of RQ1. The third research question (RQ3) is to discover the underlying reasons contributing to logistics inefficiencies in India as a logical extension of RQ2. The fourth research question (RQ4) is to assess the environmentally negative externalities of freight transport, going beyond the operational efficiency focus in RQ3. The final research question (RQ5) is aimed at discussing the potential solution concepts in practice across the world to improve the freight system performance (RQ2), logistics inefficiencies (RQ3), and negative externalities (RQ4) in India.

### Identifying and Screening of Literature

To ensure the review covered the most recent published literature on freight in India, Scopus, Web of Science, and TRID were combined with Google Scholar. The initial search used the general keywords ‘freight transportation/transport’ but this was later narrowed down to ‘India’, and subject areas ‘engineering’, ‘social sciences’, ‘environmental science’, and ‘decision science’. The search process was repeated using additional keywords of interest to this study, such as ‘logistics inefficiencies’, ‘freight traffic’, and ‘freight system performance’. Only peer-reviewed articles (including both review and original papers) were considered but these were combined with grey literature reports and government (e.g. Government of India) and international organizations (e.g. the World Bank) publications. The initial 290 unique records published between 1990 and 2020 were screened and purged down to 49. Many papers were irrelevant to the present study, as they dealt with issues unrelated to the research questions posed, despite having come up in the search exercise. The limited number of relevant papers found in the published literature underline the urgent need to focus on these research problems.

### Review Approach and Data

**Fig. 2:**
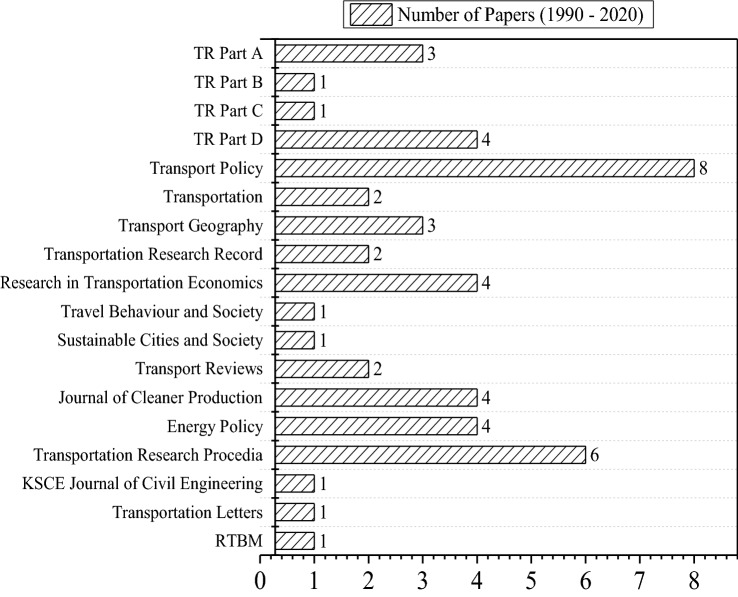
Number of papers reviewed from the literature and their respective journals

**Fig. 3:**
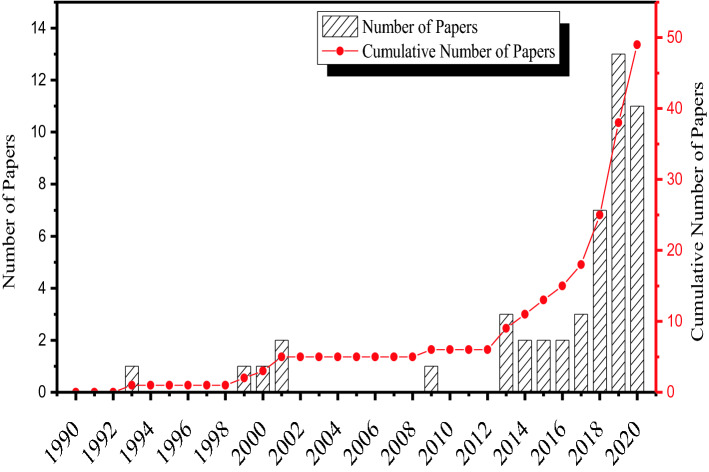
Number of papers published each year between 1990 and 2020

The papers that survived the pruning were papers published in the following journals: Transportation Research (TR) Part A, TR Part B, TR Part C, TR Part D, Transport Policy, Transportation, Transport Geography, Transportation Research Record, Research in Transportation Economics, Travel Behaviour and Society, Sustainable Cities and Society, Transport Reviews, Journal of Cleaner Production, Energy Policy, Transportation Research Procedia, KSCE Journal of Civil Engineering, Transportation Letters, and Research in Transportation Business and Management. To quantify the extent of literature relevant to freight transportation in India (RQ1), the number of papers published in each of these journals is presented in Fig. 2 and the total number of publications in each year is presented in Fig. 3. As can be seen in Fig. 3, there has been substantial growth in freight studies since 2018. This jump in the number of papers underlines the increased research attention given to this topic area in recent years. Out of the 49 papers, 41 were published in Elsevier journals (83.67%), 3 were published in Springer journals (6.12%), 3 were published in Taylor and Francis journals (6.12%), and 2 were published in SAGE journals (4.08%). The 49 papers can be categorized into four areas: (1) development of disaggregate-level freight demand estimations at seaports [5, 6] or urban establishments [7–11], (2) development of aggregate-level freight generation [12, 13] or distribution models [14], (3) design of establishment-based freight surveys [15, 16] and zoning systems [17–20], (4) analysis of freight transport parking practices [21], emissions [22, 23], expenditure patterns [9, 24, 25], and logistics sprawl [26]. Since the relevant statistics on freight system performance (e.g. modal share, logistics cost) or specific logistics inefficiencies related to India were missing in these publications, the review scope was also extended to collect aggregate-level data from publicly available sources, such as the Logistics Performance Index from the World Bank [39] and the Freight Transport Indicators from the OECD [38]. Additionally, policy briefs and reports published by government agencies in India were also referred to gain insights into the status of freight transport policies. The Indian Government National Transport Development Policy Report [27] was reviewed with the aim of capturing the policy discourse. The discussion derived from the identified literature follows the structure shown in Fig. 1. The thematic discussions on three specific topic areas are provided in the next three sections: freight system performance in India (RQ2), logistics inefficiencies in India (RQ3), and negative externalities of freight traffic in India (RQ4). The solution concepts for the issues identified in the thematic discussions are discussed in the penultimate section. The final section concludes this paper.

### Freight System Performance in India

In line with the rest of the world, freight transport is undergoing important changes in India, most of which are simply a result of market trends, and most of which are already shaping and will continue to significantly shape the way freight transport performs in the future. This section is devoted to scrutinizing these trends, putting them in context, and understanding their potential impact in the short and medium term.

### Unbalanced Modal Mix and Growth in Road Freight

**Fig. 4:**
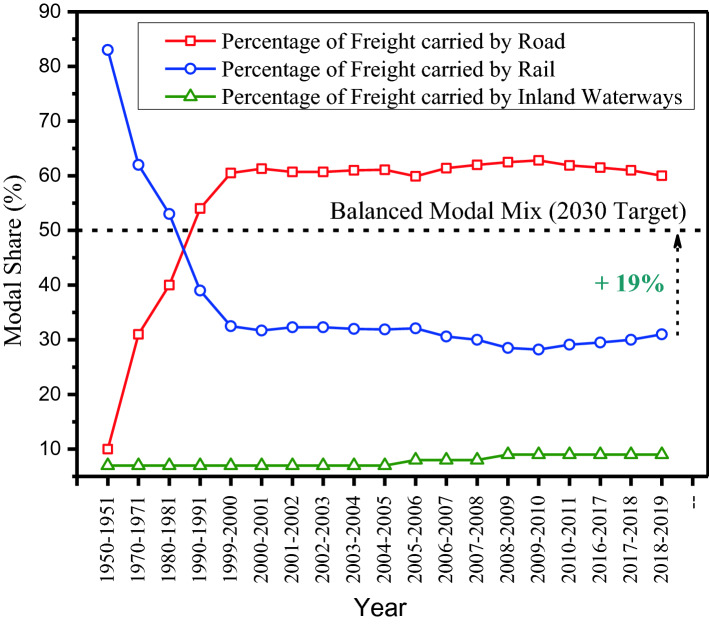
Variation in freight modal share in India over time. Source: Urban Freight and Logistics: The State of Practices in India [31]

The Indian economy is growing rapidly, partly thanks to its ongoing industrialization. One of the consequences of this is that freight movements are growing exponentially—both in the last-mile and long-haul trucking sector [27]. A major share of this demand is carried by road transport due to the flexibility provided for first-mile and last-mile logistics. The freight transport sector in India, as a result, is heavily skewed towards road transport with a modal share of 64% [28]. This compares with 75% in Europe [29] and 63% in the USA [30]. The historical trend of modal share between road, railways and inland waterways is presented in Fig. 4 using secondary data publicly available from a report published by the “Sustainable Urban Transport Project”, an organization devoted to the study and promotion of sustainable transport in urban areas [31]. The rail market share, as it can be seen, has gradually declined over the years. This trend is concerning because the economies of scale for bulk cargoes can be better achieved using a combination of railway, inland waterways, and coastal shipping. In 2018–2019, freight mode share in India stood at 27% rail, 64% road, 5% coastal shipping, 2% inland waterways, and less than 1% air (plus a 2% via pipelines for gas, water sewerage, etc.) [28]. Considering the less than optimal rail share, Indian Railways, a government entity under the Ministry of Railways that operates India’s national rail system, has set a target of having at least a 50% share of the country’s freight traffic by 2030 [32]. To achieve this, Indian Railways is investing heavily on network expansion projects and dedicated freight corridors. The strategic planning of these large-scale projects requires accurate freight demand models [24, 25, 33, 34].

**Fig. 5:**
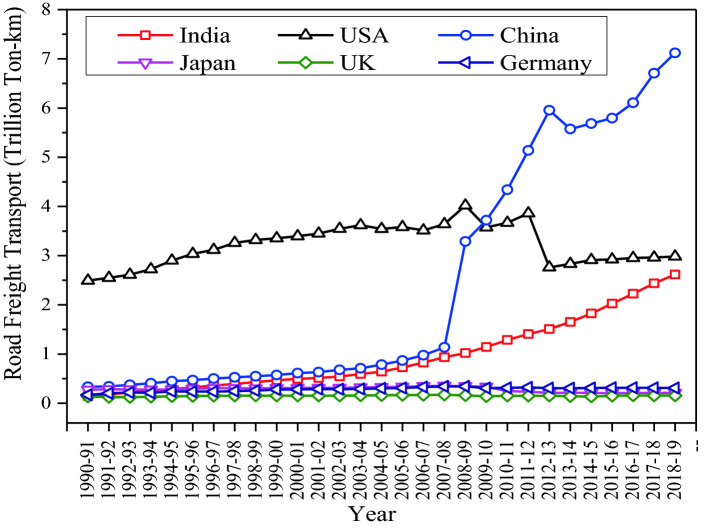
Growth of road freight transport in India and other major OECD countries. Source: OECD Freight Transport Indicators Database [38]

Achieving an efficient modal share is important for a country and the environmental impacts of different transport modes are evaluated in several studies [35]. An efficient modal share is one that maximizes volumes transported and does so at the minimum social cost, to include not just time and vehicle operating costs, but also externalities, especially noise, air pollution, climate change, caused by greenhouse gas (GHG) emissions, and accidents. For example, large, regular flows of goods with low-value density are historically suited for transport by railways, because: (i) origin/destination points tend to remain the same; (ii) commodity fragmentation can be avoided, and (iii) emissions can be minimized. Medium-valued goods are also increasingly transported by railways due to the availability of modern intermodal services around the world [36]. Due to significant economies of scale, railway or inland waterways have the potential to move these goods at a much lower unit cost than trucks with far lower GHG emissions and cost variability. A recent comparison of freight mode performance in India [37] suggests that the unit costs of moving goods is highest for road transport (2.58 INR/ton-km or 3.4 US cents/ton-km at April 2022 exchange rates), followed by railway (1.41 INR/ton-km or 1.86 US cents/ton-km at April 2022 exchange rates) and waterways (1.06 INR/ton-km or 1.40 US cents/ton-km at April 2022 exchange rates), respectively. Despite the high unit costs and road freight externalities, freight transport is road dominated in India (and other regions of the world such as Europe and the USA, as already highlighted above) because it offers greater delivery flexibility and shipment size. This trend is reflected in the growth of (road) freight transport in emerging Asian countries, such as China and India, as shown on Fig. 5. The data were collected from the OECD Freight Transport Indicators Database [38]. The figure shows a clear growth of road freight in emerging economies, in line with the growth of the freight sector in those countries, and of the economy in general. Freight flows in OECD countries of North America and Europe, on the other hand, have reached a steady-state.

Road freight transport offers lower transit times and higher reliability, making it better suited for transport of perishable goods and commodities with high value density [17]. The other operational advantages of using trucks include saving in packaging costs, ability to track and trace cargoes, door-to-door serviceability, and ability to schedule the delivery. An effective mode share of a country should thus concomitantly satisfy two criteria: (i) minimizing transport costs and (ii) meeting the operational requirements of shippers. While there is no consensus on the “ideal” modal mix for freight transport, India’s geographical features (extensive coastlines, predominance of hinterland economic activity, longer length of hauls) and the need to reduce freight emissions point in favour of rail transport.

### Logistics Cost, Performance and Global Benchmarks

**Fig. 6:**
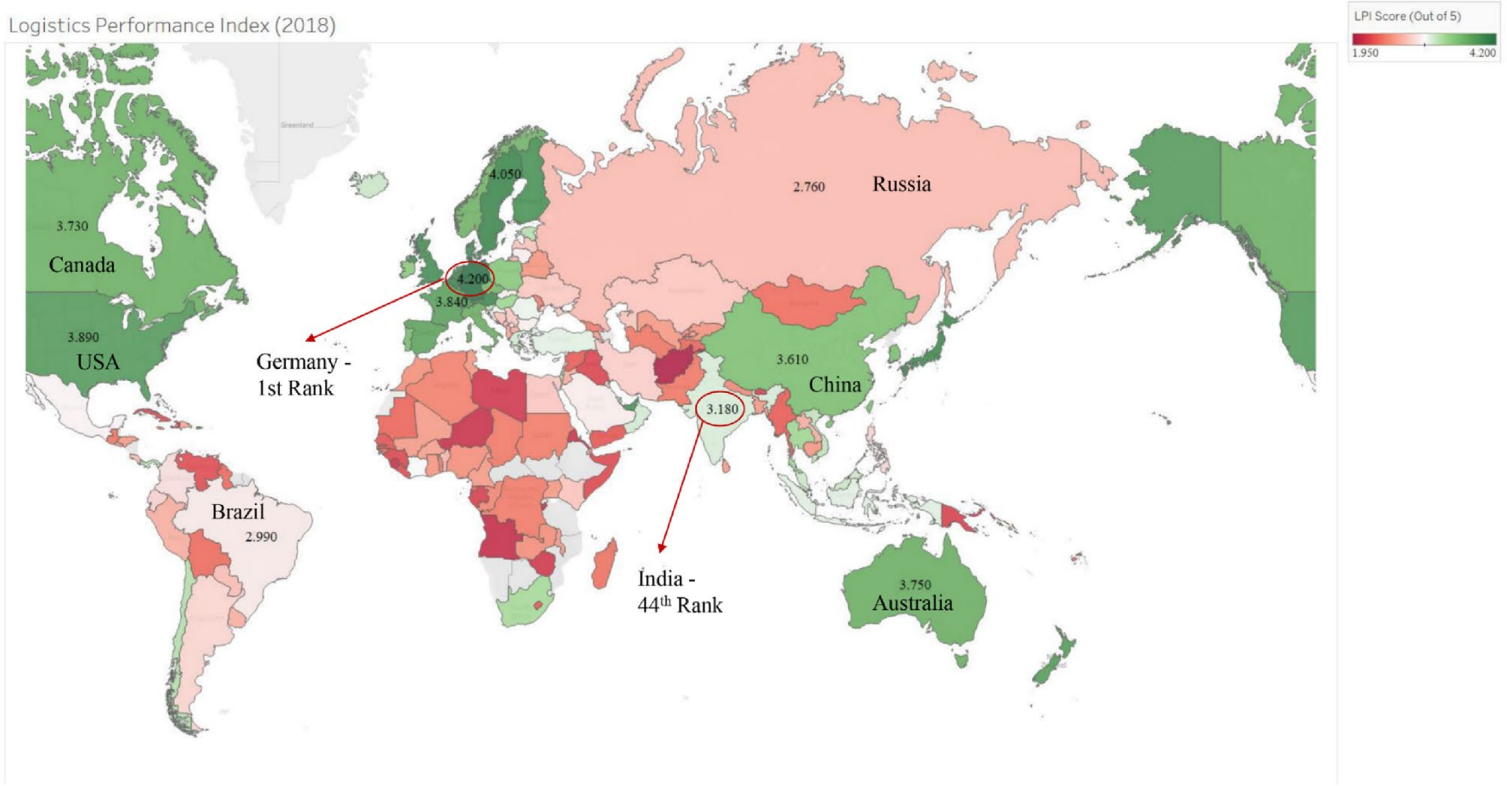
Global variation in logistics performance index. Source: Logistics Performance Index [39]

Logistics costs are a significant component of total trade costs. The high logistics costs constrain the competitiveness of the economy and are often the result of shortcomings (physical, regulatory, or institutional) in the transport sector. Nearly one-third of India’s logistics costs (~ 4% of GDP) are attributed to inefficiencies in infrastructure. An important logistics measure that can be used to compare the performance of India’s logistics system against its competitors is the Logistics Performance Index (LPI) produced by the World Bank [39]. The LPI scores are based on data on six dimensions of trade: customs efficiency, infrastructure quality, ease of transporting international shipments, logistics quality and competence, trackability of consignments (also called tracking and tracing), and delivery timeliness [39]. The World Bank uses the LPI to rank countries [39], and a summary of this ranking, relevant to the present paper, is presented in Fig. 6. As it can be seen in Fig. 6, the logistics infrastructure in India lags behind that in Germany, the USA, the UK, and China. Jumping up the LPI rank, as currently proposed [40], will require a fundamental reorientation in the way logistics infrastructure caters to freight demand in India.

**Fig. 7:**
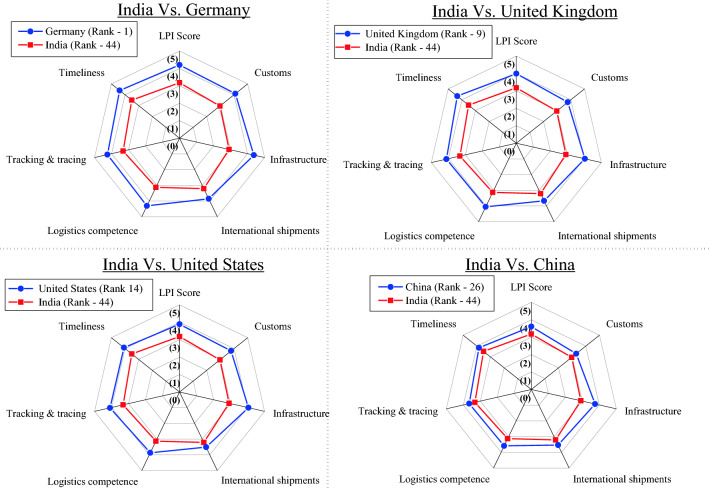
Pairwise comparison of LPI between OECD countries and India. Source: Logistics Performance Index [39]

Pairwise comparisons of LPI scores between major OECD countries and India are given in Fig. 7, to highlight the deficiencies across different aspects of logistics.

### Changes in Logistics Strategies and Freight Needs

One important challenge in the freight sector is linked to the changes that have taken place and continue to take place in the context of E-commerce [41]. The purchasing options of consumers in the past were limited to retailers in the city centre, whereas these are now competing with wide-ranging options provided by online retailers. To maintain a competitive edge in the market, shops are increasingly adopting just-in-time inventory practices, which result in stocks being kept to a minimum. Another noticeable change is the increased requirement for better logistics outsourcing service levels [9]. Many customers expect delivery within 24 h after placing an order [10]. Retailers are forced to respond and adapt to changing consumer requirements or risk losing them to the nearest competitor. Compounding this challenge is the consumer experience, which has become highly personalized and specialized, thanks to the digital transformation that has taken place since the early 2000s. This implies more customized orders, stricter quality controls, tighter compliance standards, shorter delivery windows and an overall intolerance to delays in shipments. The role of “fulfilment centres” became increasingly important over the first two decades of the 2000s, and is now a component in many supply chains, with a prime example being Amazon. The changes in logistic strategies of many stores (and warehouses) and the expectations from end consumers have had significant impacts on the demand for freight transport as follows: (i) there is a higher demand for goods, (ii) there are higher service levels, and (iii) shipment sizes tend to be smaller than they used to be.

### Diversification of Freight Flows

**Fig. 8:**
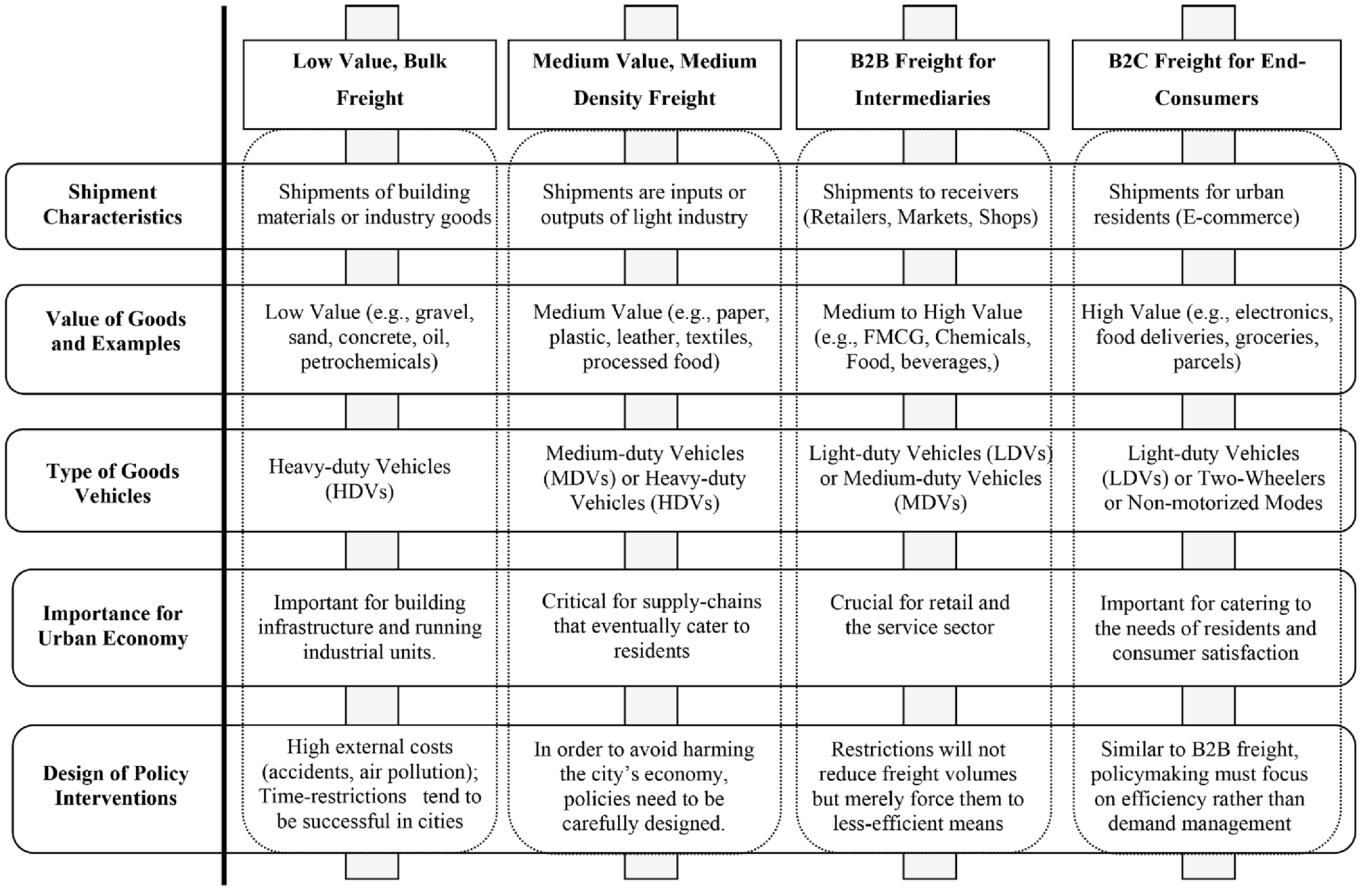
Type of shipments transported by urban freight. Source: authors’ own conceptualization based on freight flow categories discussed in the report by the Ministry of Housing and Urban Affairs and Rocky Mountain Institute [42]

**Fig. 9:**
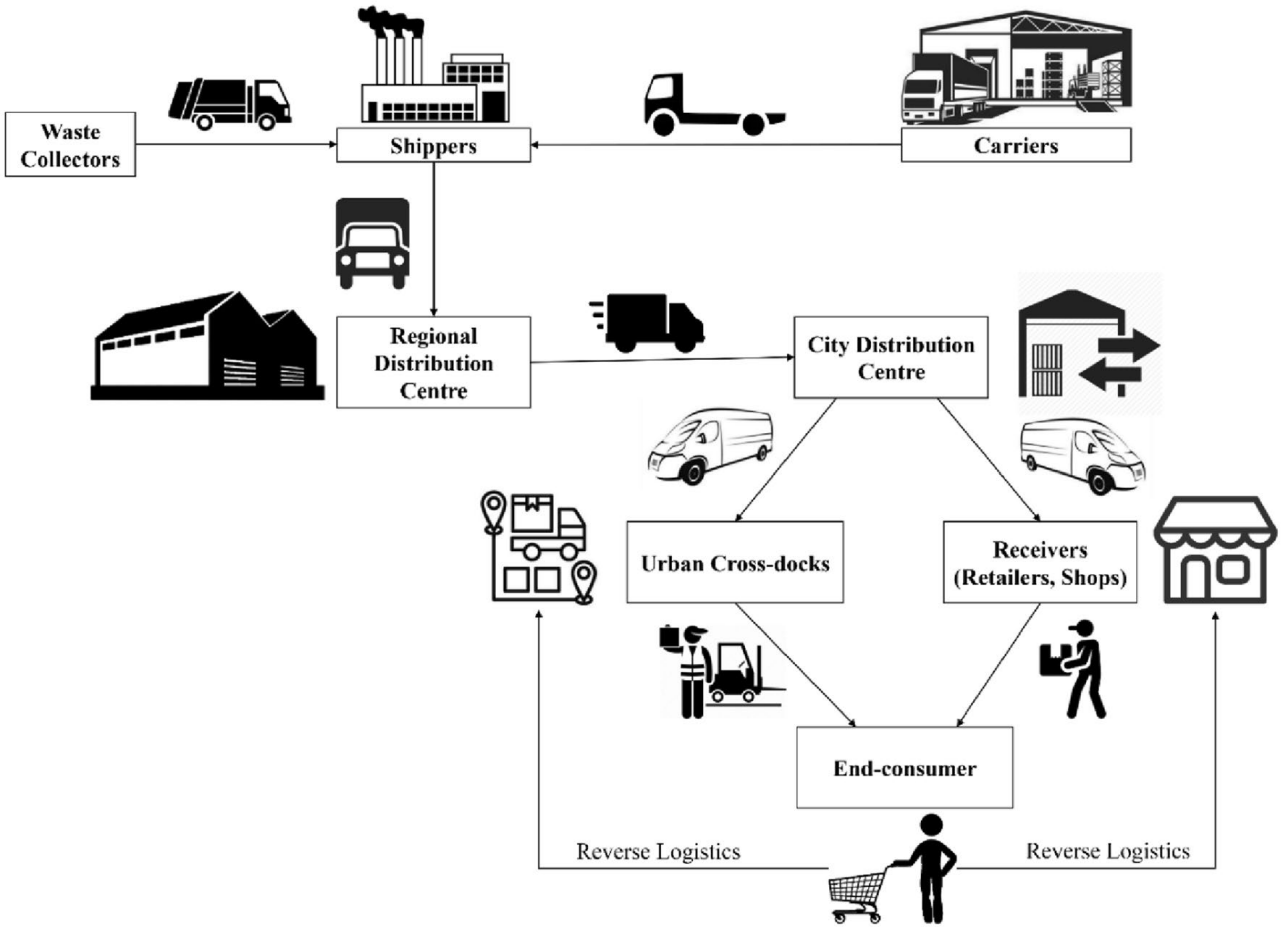
Conceptual layout of urban supply chains. Source: authors’ own conceptualization, extending the layout in Pani and Sahu (2019b)

There are different types of freight flows. These are depicted in Fig. 8, based on the typology suggested in a report by the Ministry of Housing and Urban Affairs and Rocky Mountain published in 2019 [42]. As shown in Fig. 8, there are four different types of shipments: (i) low-value, bulk freight (LVBF), (ii) medium-value, medium-density freight (MVDF), (iii) business-to-business freight (B2BF) for urban consumption, and (iv) business-to-consumers freight (B2CF) for urban residents. LVBF refers to shipments of construction materials (concrete, sand, gravel) and industrial goods (oil and petrochemicals). LVBF accounts for a significant share of freight shipments, especially in cities where the majority of the infrastructure is still in the process of being built. These low-value shipments tend to be shipped in large quantities (and heavy-duty vehicles), which cause high external costs. The next spectrum of shipments refers to MVDF, which are inputs or outputs of light industry (raw material oriented and less capital intensive), such as, for example, paper products, plastic products, leather, and textile products. The B2BF shipments are typically directed towards retailers so that they can be sold to urban residents. These shipments include fast-moving consumer goods, such as, for example, food products, beverages and pharmaceuticals, and they are typically stocked on the shelves of consumer stores. B2BF shipments are characterized by a high frequency of trips, although they are typically transported in light or medium-duty vehicles. Restricting the movement of B2BF shipments is generally contentious, because B2BF shipments directly cater to the needs of urban residents. Instead of reducing the freight volume, restrictions are typically found to force the shippers to move freight in less efficient ways. B2CF shipments typically are of high value and have specialized handling and delivery requirements (e.g. food deliveries, document packages, parcels). These types of freight are transported in light-duty vehicles, vans, two wheelers or even by foot, directly to the end consumer. Formerly a relatively small segment of urban freight travel market, B2CF shipments have become critical in urban logistics with the rise of E-commerce. Much like B2BF shipments, B2CF shipments cater to urban residents and policy interventions should focus on efficiency rather than demand management. While these diversifications are unique in each supply chain, a general conceptualization of urban supply chains is presented in Fig. 9.

B2CF shipments have experienced an important growth in the last few years, mainly due to increased Internet penetration. In the organized retail market, the share of online purchases was 25% in 2019, with forecasts predicting it could reach 37% by 2030 [43]. The presence of online platforms also enables consumer-to-consumer online markets; this form of E-commerce is rising in popularity [44]. Due to the influx of online platforms, many traditional retailers feel the need to participate in E-commerce as well, further increasing the urban freight flow levels. Many retailers are choosing to sell goods held in their inventory through E-commerce and Omni-channel delivery systems to offer better options to consumers [45]. In the context of increasing B2CF flows, reverse logistics of goods are also becoming more important. These streams involve not only the return and exchange of goods purchased online, but also services such as waste collection. As it can be seen in Fig. 9, shippers also produce additional freight activity in the form of waste and “reverse logistics” of returns and exchanges. Policymakers should therefore customize policy interventions to different types of freight traffic. For instance, shippers or retailers can offer customers (end node of supply chains) unique value by incentivizing reuse of raw or finished materials through a seamless “return” policy. By creating these feedback loops in supply chains, cities can transition towards a circular economy with significant societal and economic benefits [46]. Classifying the type of goods entering the city through different supply chains can be the first step for understanding the diverse needs of the freight market segments and, in turn, examining what challenges they present. Existing freight studies in India have largely focused on freight flow, and limited attention has been given to reverse logistics and service activities [9, 16, 47].

### Infrastructure Investment and Mobility Studies

**Fig. 10:**
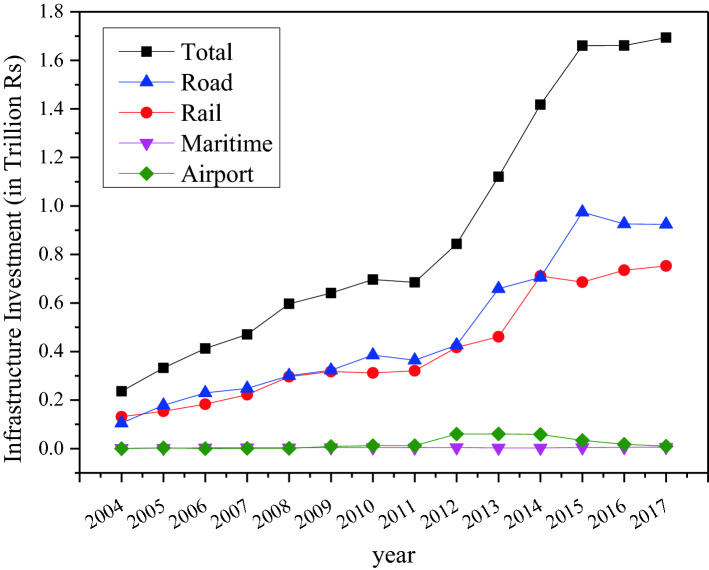
Growth in infrastructure investment. Source: OECD Infrastructure Investment Database [49]

Productive investment on freight transport infrastructure is vital for improving the freight system performance and, in turn, enabling seamless deliveries and pick-ups of goods in urban areas [48]. An integrated approach to infrastructure spending, with investment schemes driven by transport policy goals that are coordinated with land-use and industrial development objectives, is critical for India. Since much of the freight movements have a destination in cities where ports or airports are located, infrastructure investment needs to be prioritized in those cities and regions, especially as freight vehicles share road space with passenger traffic. A comparison of infrastructure investment in India over the period 2004–2017 is presented in Fig. 10.

There have been several initiatives for increasing capacity, such as for example, the construction of dedicated freight corridors (DFCs). DFCs are expected to ensure that long-haul freight demand is catered efficiently in existing trunk routes on the eastern and western corridors (Howrah-Delhi and Mumbai-Delhi), which are currently saturated with line capacity utilization of 115%–150% [27]. The diversion of freight traffic from the long-haul trucking sector to DFCs on truck routes is expected to decongest the existing highway network for passenger movement. However, appropriate transport supply improvements require a demand assessment toolkit which is still missing for India [18].

### Logistics Inefficiencies in India

There are a number of inefficiencies in both freight transport and freight policies in India. These inefficiencies can be broadly categorized into four areas: (i) long-haul trucking, (ii) last-mile logistics, (iii) freight distribution (inventory level), and (iv) policies and regulations. Reducing these inefficiencies will reduce the generalized cost of moving goods and the externalities of road transport. It will also improve the satisfaction of urban residents.

### Inefficiencies in Long-Haul Trucking

The inefficiencies in trucking costs are driven by three factors: (i) avoidable running costs created by empty backhaul of trucks, (ii) usage of trucks with reduced fuel economy and (iii) insufficient fleet size and mix of logistics providers, which lead to inefficient utilization of trucks for forwarding shipments. The root cause of these inefficiencies is related to the inability of trucking firms to achieve economies of scale, thereby resulting in low productivity and efficiency. This is partially linked to the rise of small trucking firms, which attempt to reduce logistics costs through overloading, service violations and poor maintenance. These unlawful logistic operations artificially lower the price of trucking services and make the traditional carriers with large efficient fleets unable to continue operations. Large trucking firms, on the other hand, can achieve economies of scale through efficient dispatching and scheduling, which is critical to increasing fleet utilization and reducing empty running. Another important contributor towards inefficiency is the suboptimal load size observed in emerging countries [37]. The highways in India are also inadequately maintained, inconsistent in road width and heavily congested [27]. These infrastructure shortfalls underline the need for targeted capacity increase to improve the inefficiencies in long-haul trucking.

### Inefficiencies in Last-Mile Logistics

Last-mile logistics involves delivering packages to end consumers or retail shops in urban centres. It typically follows different trip patterns, uses different vehicle types and has a different spatial extent of travel, compared to long-distance trucking. Due to the nature of multi-stop delivery tours carried out in last-mile operations, priority is given to maximizing the amount of freight delivered in an average tour. This is in contrast with the priorities given to achieving improved shipment size in long-distance trucking. The importance of last-mile logistics, despite being the shortest link in supply chains, stems from the fact that it constitutes up to up to 13%–72% of total logistics costs in many supply chains [50], and up to 55% in supply chains involving E-commerce [37]. The variation in costs is because of several potential causes of inefficiencies that exist in last mile, as explained below.

- **Fragmentation of receivers:** In India, as in many emerging markets, the demand for freight can be somewhat fragmented. The reason for this fragmentation is the informal, even impulsive, ordering behaviour of “nano stores” [51]. This ordering behaviour affects the performance of the whole supply chain, as it triggers actions upstream in the supply chain [51].
- **Fragmentation of carriers:** Planning delivery tours is a complex optimization problem, which needs to maximize delivery quantities, minimize time and distance whilst reaching all destinations, considering delivery windows and traffic patterns/congestion**.** Logistic providers in developing countries like India often lack the fleet size and technical tools to dispatch delivery trucks on optimal tours.
- **Fragmentation of delivery points:** In the era of E-commerce and highly personalized freight orders, fragmentation of delivery points is an important barrier to last-mile logistics efficiency. The discretization of delivery points is a more pronounced issue in urban areas than the fragmentation of receivers. This is a problem common to both developed and developing countries, but in countries like India, the impacts are more evident, probably because of the higher traffic congestion levels that prevail in most cities.
- **Logistics sprawl:** Due to high land values in cities, warehouses and distribution centres tend to migrate towards the suburbs. This phenomenon, known as “logistics sprawl”, increases the duration of delivery tours and the resultant traffic increases congestion levels, both going into and out of the cities [52]. Transportation is intrinsically linked to the urban growth phenomenon and the associated logistics sprawl [53, 54]. Another implication of logistics sprawl is that it reduces the number of delivery points accomplished in a single tour. The evidence for logistics sprawl in major Indian cities is already available for industry sectors such as the timber market [26].

### Inefficiencies in Freight Distribution

Inventory is a critical element of logistics costs, as it requires facilities for storage and holds up the working capital of firms (i.e. receivers) in a freight system [55]. To reduce these costs, firms typically attempt to minimize inventory levels without compromising their ability to serve end consumers. Due to uncertainty in lead times (i.e. time taken by shipper to deliver goods), excess inventory costs are typically incurred by shippers in the freight system, thereby increasing the overall logistics costs. The reduced reliability in transit times leads to higher buffer stocks to guard against uncertainty. The highly fragmented and inefficient distribution system poses major challenges to buffer stock reductions. Another challenge is the limited digitization of links connecting the stakeholders in a freight system, which restricts the ability of retailers to reduce cycle stock (i.e. the inventory held in shelves to satisfy normal sales demand). The ability to implement just-in-time (JIT) ordering practices that can accomplish reductions in cycle stock hinges on two factors: (i) digital capabilities to track inventory drawdown and (ii) digital links to distribution centres and supplies to avail dynamic replenishment of products. Due to limited advances in JIT systems in India, efforts to reduce the total amount of inventory in the distribution system and the amount of inventory lost have not been very successful [37].

### Inefficiencies in Policy Framework

Policies intended to reduce the negative externalities or inefficiencies from freight can actually backfire and yield the opposite result. The National Urban Transport Policy (NUTP) in India acknowledges that freight traffic will grow substantially [31, 56]. Timely and seamless freight movements are also mentioned as a priority for the economic development of the country [31, 56]. The freight-related policy measures recommended in the NUTP report can be summarized as follows: (i) using off-peak hours for freight deliveries, (ii) restricting the entry of heavy-duty trucks into cities during daytime, (iii) building bypasses through public–private partnerships so that long-haul trucks can go around the city, instead of adding to the city traffic, (iv) reorganizing land use by locating wholesale activities in the periphery of cities, along the interstate highways, rather than in city centres, (v) building truck terminals and parking facilities outside the city limits to encourage the shifting of wholesale activities, (vi) provisioning parking space at appropriate locations for on/off street with the use of intelligent transport systems, (vii) planning ring roads to relieve traffic congestion in central areas, and (viii) implementing auto-fuel policies that call for tighter emission regulations and fleet upgrades [31, 56]. Following the recommendations of the NUTP report [31, 56], some cities, including Ahmedabad, Bangalore, Hyderabad, and Kochi, have set up committees, known as Unified Metropolitan Transport Authorities (UMTAs), charged with the mission of integrating the functioning of agencies associated with passenger and freight mobility. These top-down policies may deliver positive impacts and help achieve more efficient freight movements in urban areas.

Another policy that has been suggested is the implementation of time-based or cordon-based restrictions. These restrictions have been introduced in some cities in India [7]. They can entail, for example, banning vehicles exceeding 7.5 tons during specific time periods of the year or specific times of the day [7]. Delhi has also banned non-destined transiting trucks (heavy, medium or light-duty vehicles) from passing through certain regions in Delhi [23] and has imposed entry time restrictions to freight destined for Delhi. Shifting freight travel into times of minimum residential use can force deliveries at night, greatly increasing the share of last-mile cost in total logistics cost. Furthermore, it can, and it has, resulted in good deliveries by vans or three wheelers, which are not subject to bans, and this can, and indeed has, in turn, increased overall traffic.

In addition to the above, the imposition of pollution taxes can help freight face the environmental costs they cause. Delhi, for example, introduced a pollution tax in 2015, payable by trucks passing through Delhi [23]. The tax is 700 INR and 1400 INR (USD 9.24 and USD 18.49 at April 2022 exchange rates) for light-duty vehicles and heavy-duty vehicles, respectively. Reorganizing land use by moving wholesale markets to outer town suburbs or satellite towns can help reduce the pressure from freight movements. Mumbai did exactly this to reduce traffic levels in the congested south part of the city. Another initiative is that of Urban Consolidation Centres (UCCs) [57]. These schemes aim to reduce the number of goods delivery vehicles in urban areas by consolidating multiple shipments at centres located in the city periphery [58]. There are several informal examples of such centres in India, especially in the perishable product sector (e.g. Azadpur vegetable market, sabzi mandi in Delhi). Another example of consolidation is the ITC e-Choupal project in which internet-based kiosks reach out directly to farmers and eliminate the middleman in agri-business supply chains [31]. Finally, many cities, such as for example Chennai, are planning to have truck terminals and parking zones on the city periphery [31]. However, there are several institutional, practical and legal barriers for long-term success in UCCs implementation, as reported in some European cities like Oslo [57].

Despite the publication of the NUTP and the setup of UMTAs in some cities, freight transport policy in India is still in nascent stage [42], in contrast with passenger transport policy. Save for the policy initiatives aimed at increasing capacity and building facilities (truck terminals, consolidation centres), freight policies in India have largely been restrictive in nature [23]. A scenario building approach, perhaps taking into account individual perspectives [59], has the potential to yield participatory decision-making outcomes related to freight policies.

## Negative Externalities of Freight Traffic in Indian Cities

The negative externalities from freight traffic in India are only expected to increase in magnitude, bearing in mind the trends mentioned in previous sections. Negative externalities from freight can be defined as the costs imposed by freight on freight and other traffic, and society in general. These costs are not borne by those causing them, and are not reflected in any economic transaction (i.e. when the good is produced, transported or consumed). These external costs can be broadly categorized into three [60, 61]: (i) environmental impacts, (ii) social impacts and (iii) economic impacts, as explained below.

### Environmental Impacts

The environmental impacts from freight transport include air pollution, climate change caused by GHG emissions, noise, and water pollution. A multimodal emission assessment shows that the emissions from transport are expected to grow by 4.1–6.1% per year, leading to an increase of seven times by 2050 [62]. Air pollution is caused by emissions of particulate matter (i.e. microscopic solid or liquid particles in air), carbon monoxide, ozone and hazardous air pollutants such as benzene, which causes cancer and other serious health effects. Most trucks run on diesel, which is more polluting than petrol. Climate change is caused by GHGs. Excessive noise can negatively impact human health, disturb sleep, and cause cardiovascular and psychophysiological problems [63]. Most of the external costs from trucks in Europe come from noise [64, 65]. Water pollution can result from freight transport when there are spills, leakages, or disposal of cargo material in water bodies. Although freight traffic constitutes merely 3% to 15% of total traffic in urban arterials and expressways [66, 67], it is estimated to be responsible for up to 50% of road transport emissions [68]. In the case of noise pollution and vibration hindrance, in general, road freight has a much larger impact than cars [64, 65]. Compounding these impacts is the fact that freight trucks used for urban deliveries are generally older and more polluting than trucks used for long-haul shipments [69]. Finally, land-use changes associated with freight flow and transport infrastructure development are an increasing source of concern as they can cause visual intrusion on environmental landscape, and destruction of habitats and species loss.

### Social Impacts

**Fig. 11:**
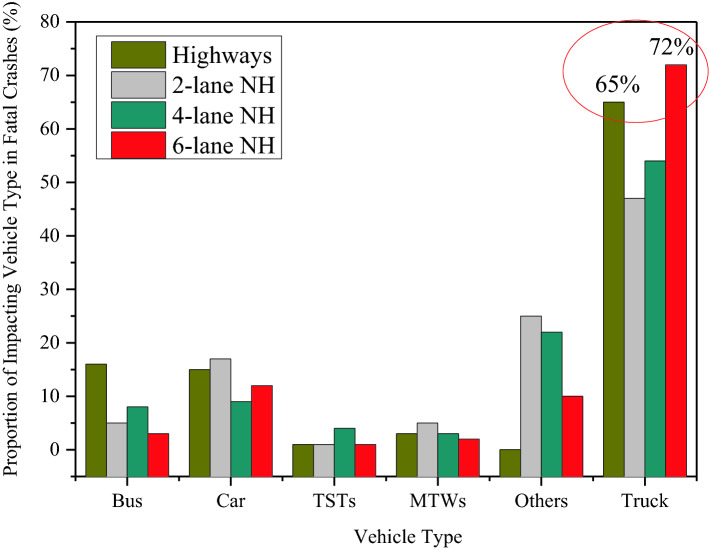
Proportion of impacting vehicle type in fatal crashes (2015–2018). Source: Transportation Research and Injury Prevention Report, Table 10 [70]

The main negative externality from freight with social impacts is accidents. A significant share of road accidents can be attributed to trucks, as shown in Fig. 11, based on crash data published by the Transportation Research and Injury Prevention Programme at the Indian Institute of Technology in Delhi [70]. The vehicle types include motorized two wheelers (MTWs), three wheel scooter taxis (TSTs), buses, cars, trucks, and others. As it can be seen in Fig. 11, 72% and 65% of fatal crashes in six-lane national highways and urban highways are associated with trucks as one of the impacting vehicles.

### Economic Impacts

The main externality from road freight that has economic impacts is congestion. Congestion caused by road freight has become a common problem in cities around the world [71]. In Europe, most of the external cost from trucks comes from congestion (and noise) [64]. Trucks take between two and four times the road space that cars take, and their speeds also tend to be lower. In addition, due to scarcity or inadequate configuration of loading or unloading bays/zones, freight trucks often double park during their delivery tours [72], thereby blocking the road for other vehicles. Traffic congestion has substantial negative impacts in terms of reduced productivity and wasted fuel. A high-level estimate of the economic loss resulting from congestion in major cities in India is over 22 billion USD per year [61]. Two conflicting interests emerge regarding congestion—public authorities aim to reduce freight traffic to improve the attractiveness of their city to residents as well as tourists, while private companies seek to operate at lowest cost with quick deliveries to satisfy consumers’ expectations in a highly competitive market [61]. The regulations and restrictions brought by public authorities can cause “detour” of delivery vehicles through narrow streets and unsafe delivery areas with low vertical clearance, further exacerbating traffic congestion [73].

## Solution Concepts for Sustainable City Logistics

Considering the past trends, future projections of freight movement in India, a portfolio of solution concepts needs to be proposed and implemented to address negative externalities and inefficiencies in freight transport. We provide a broad overview of these solution concepts in this section and do so under four categories: (i) physical assets such as infrastructure and equipment, (ii) technology and operations, (iii) policy and regulations, and (iv) logistics-driven changes. The first two classes of solution concepts are part of long-term planning and require significant investment and changes to transport infrastructure. The latter two classes are part of short-term planning and they aim to reduce the impact of freight transport within the existing expanse of transport infrastructure. These categories of solution concepts are explained in the next subsections.

### Infrastructure Solutions

These solutions include improving the quality and capacity of the road and railway networks and providing multimodal hubs and warehouses. Analyses of commodity movements and freight demand are critical decision-making tools for provision of infrastructure solutions. These solutions are imperative for developing a balanced modal mix and reducing the overall logistics cost, as explained below.

- **Improving freight distribution and last-mile logistics:** The infrastructure facilities that need to be provided to improve freight distribution and last-mile logistics in urban areas are: (i) curb-level parking infrastructure and loading bays, (ii) exclusive truck lanes and dedicated routes, (iii) urban freight consolidation centres, and (iv) urban logistics spaces and (v) smart lockers. Parking and loading bays are critical for reducing the cruising time for truck traffic since delivery locations in urban areas often lack parking infrastructure. As a result, the inability to find an unloading spot or off-street parking lot leads to double parking and congestion [74]. Development of reversible lanes (off-peak reorganization of lanes in dense business districts), developmental lines and land-use ordinances are some of the effective solutions for improving parking efficiency [72]. Exclusive truck routes help for “detouring” freight deliveries away from residential areas in urban areas. These routes need to be designed for anticipated truck traffic levels in terms of vertical clearance, turning radii, sight distances and gradients. Provision of exclusive truck routes helps to streamline freight traffic in such a manner that the operational efficiency of other roads can also be improved. UCCs allow for greatly enhanced loading and routing efficiency in last-mile logistics efficiency. UCCs implementation can reduce freight travel by up to 50% in urban areas [37]. Urban logistics spaces (ULS) present a less intrusive way of achieving shipment consolidation than UCCs. Logistics operators and shippers typically welcome ULS compared to UCCs since the former are perceived to cause less disruption to lead times and delivery frequency. Smart lockers, or pack stations, are banks of lockers placed in activity centres such as transit stations, malls, grocery stores, to allow end consumers to collect their orders during their daily activity travel pattern, instead of taking delivery at home locations.
- **Achieving a balanced modal mix:** There are two complementary targets in the roadmap for achieving a balanced modal mix in a country like India where rail freight is having a suboptimal share. One is to look at potential solutions that can foster a modal shift towards rail transport and the other is to facilitate better intermodal transfer between road and rail [75]. The former category of solutions includes the following: (i) increasing rail network capacity, and (ii) resolving gaps in rail network connectivity. The latter category of solutions includes developing intermodal logistic parks in tandem with dedicated freight corridors, and/or promoting double-stack clearance (stack containers one above the other) of intermodal corridors.
- **Reducing inventory costs:** Two major solutions exist for reducing inventory costs, a major component of total logistics costs. The first is to improve the quality of warehousing, and the second is to reorganize warehouses to optimal locations. As for the first solution, the quality of warehousing can be improved by investing on automation, cross-docking facilities and refrigeration systems.
- **Improving trucking efficiency and productivity:** The most important solution to improve trucking efficiency is to ensure that the current highway network keeps in pace with growing freight demand. Another avenue is to standardize logistic practices (e.g. harmonization of pallet and truck standards) and inventory data (e.g. inventory management for better dispatching of trucks).

### Technological Solutions

To enable logistics chains, reduce costs, and improve services for customers, freight systems need to be enriched in various technologies. Digitization, coupled with adequate technological support and targeted investment schemes, can integrate the supply chain from demand forecasting stage to shipment consolidation, truck routing and dispatch scheduling [76]. The potential solutions through these technological, digital and operational advancements can be explained on seven fronts: (i) developing more accurate demand forecasting models through enhanced inventory visibility, (ii) automation of warehouse processes, (iii) deploying inventory data insights in distribution network design to deal with demand volatility, (iv) implementation of just-in-time inventory systems and fostering lean ordering behaviour among establishments, (v) achieving efficiency in truck routing and dispatch through real-time information, (vi) implementing intelligent transport systems (ITS), such as weigh-in-motion systems, delivery space booking systems, and route planning systems, and (vii) promoting carrier collaboration and accomplishing higher levels of operational efficiency through “Internet of Things” applications. These technological solutions are increasingly explored by the new third-party logistics providers, freight forwarders, and trucking companies emerging in the Indian market. For instance, driver relay models are increasingly adopted to reduce the continuous driving time of truck drivers to less than a day, and in turn reduce the turnaround time on long-haul routes (eliminating the driver idling time in the prior operational models). Increased adoption of location tracking solutions and growing presence of fulfilment centres in Indian cities have been helping the emerging logistics companies to eliminate the inefficiencies in the traditional hub-and-spoke model of delivering parcels. By utilizing distributed delivery models (i.e. each arc in the network acting as a hub and a processing centre by itself), the delays in routing the shipments through a hub before reaching the spoke can be avoided with the help of technology. As a result, most of the emerging logistics companies are positioning themselves as supply chain enablers with their own in-house order management systems. The challenges faced by small fleet owners have also come to the focus of the emerging market players in the logistics space with a vast number of software-as-a-service (SAAS) companies working towards hassle-free truck bookings and real-time vehicle tracking. The ongoing efforts as a part of Government of India’s Gati-Shakti national master plan to develop a unified logistic interface platform (ULIP) are expected to further accelerate the efforts of trucking companies and SAAS providers to reduce the overall costs of logistics and time in India.

### Policy Interventions

Policy interventions play a crucial role in translating the first two solution classes into action, both in terms of energy demand and economic consequences [77]. Government departments, such as the Ministry of Shipping and Logistics, can employ a wide-range of policy measures, ranging from taxation instruments (e.g. fuel taxes, excise taxes and tolls) to financial incentives (e.g. tax rebates for supporting greener modes, capital grants) and regulation orders (e.g. vehicle design, entry time, emission standards), as explained below.

- **Taxation:** Apart from the typical taxes levied on petroleum products (24–25% by the central government and 20–25% by state governments), additional charges such as the ‘green surcharge’ (up to INR 2/litre, or US cents 2.6/litre at April 2022 exchange rates) exist in India, although they do not include diesel vehicles. Introducing such differential charges for trucks can favour a switch to alternate modes, such as electric trucks or rail and water. The political challenges of introducing a carbon tax in developing countries are well known [78] and require more coordinated efforts in the future to foster a nationwide change to low carbon logistics.
- **Financial incentives:** The financial support provided by the government varies from initiatives such as off-hour deliveries to incentives for shifting to greener freight modes such as electric trucks. The extent of financial support depends on external factors such as (i) differences in service and infrastructure ownership, (ii) competition policy, (iii) nature of freight market, and (iv) regulations governing financial aid from governments. Incentives also include capital grants to develop rolling stock or vessels for intermodal transfer and terminal development. Depending on the contribution to achieving government-level goals of sustainability, many services and infrastructure provisions can avail discounted infrastructure payments, operating subsidies or revenue supporting grants.
- **Supply chain digitization:** As discussed in the previous section, digitization of supply chains and enforcement strategies can improve trucking efficiency. For instance, weigh-in-motion (WIM) implementation helps to penalize shipments that are exceeding the allowable limits and helps to identify the defaulters in the freight system; this also allows for effective checkpost clearance since trucks do not need to stop for inspections. The introduction of the electronic way (e-way) bill under tax-reforms like GST has improved clearance times across various states in India.
- **Zoning for freight operations:** Land-use planning needs to develop designated locations for intermodal facilities such as inland container terminals, which can reduce urban congestion and foster a shift towards smaller commercial vehicles [18]. For this purpose, premium city space may need to be made available for logistical development near significant freight generating areas [19]. Another important aspect of land-use planning is to encourage spatial clustering of manufacturing firms that can achieve economies of density, which can lower transport costs down and improve delivery efficiency.
- **Zoning for logistic sprawl:** Due to increasing land values in city cores, logistic land uses tend to locate farther from the city centre [79]. This sprawl of logistic facilities increases daily truck kilometres travelled, as well as congestion on urban arterials. By developing an efficient zoning policy for reserving suitable land uses in city centres (e.g. creation of urban logistics spaces or ULS near major retailing chains), logistics sprawl can be reversed. By bringing ULS to city centres, urban residents can also benefit in terms of superior access to goods and services.
- **Low emission zones:** Low emission zones (LEZ) are geographic areas that limit access to those vehicles meeting certain emission standards [80]. The purpose of LEZs is to restrict or put a price on the most polluting vehicles if they enter areas in close proximity to urban residents. LEZs are typically proposed in areas where air quality levels are hazardous to society.
- **Delivery vehicle restrictions:** These are among the most common policy responses taken by public authorities when freight traffic is sharing the same right of way with passenger traffic [81]. Implementation of these restrictions without the provision of ULS or UCCs are found to have negatives impacts on the regional economy. Besides, these restrictions often turn counterproductive due to increased delivery activity using small vans and three-wheelers. In the aftermath of the COVID-19 pandemic, delivery vehicle restrictions have received increased attention due to the rising delivery activity in residential areas, largely driven by the emergence of grocery and food delivery companies. As the delivery start-ups are primarily focusing on faster deliveries and increased convenience for consumers, fulfilment/distribution centres are being deployed in the middle of dense urban neighbourhoods and delivery drivers are incentivized to achieve 10-min or 15-min delivery windows. The traffic safety concerns resulting from these delivery vehicles have thus been receiving notable coverage in the newspapers, underlining the requirement of data-driven delivery vehicle restrictions and centralized self-service delivery lockers as a mitigating solution.

### Market-Driven Solutions

The final class of solution concepts is related to the market-driven changes that can be implemented in the freight transport sector. These solutions include three broad categories: (i) technological advances, (ii) crowd shipping on transit (COT) programs, and (ii) planning and cooperation initiatives, as explained below.

- **Electric and autonomous freight vehicles:** A number of technological advances in the freight transport sector have been made in the field of vehicle technology [82–84]. These advances are increasing the fuel efficiency of trucks and reducing emissions through emission filters. Continuous improvements are being made with respect to noise reduction and safety hazards. Furthermore, electric trucks (ETs) and connected and autonomous trucks (CATs) are becoming more scalable and viable alternatives relative to diesel powered trucks [85–87]. Analysis of the passenger transport sector already shows that the policy push for e-vehicles will only reduce GHG emissions if the electricity generated to power these vehicles is produced in a clean manner, i.e. the electricity generation mix needs to have a large share of renewables [88]. Fostering the replacement of traditional truck fleets with ETs and CATs through incentive schemes and tax reductions will significantly reduce the negative externalities of freight transport. Autonomous delivery robots (ADRs) are another emerging technology in retail and are projected to be a crucial step towards low-carbon last-mile deliveries [89]. With various tests underway, researchers believe that ADRs could revolutionize the system and reduce delivery costs by 80% to 90%. Although the current state of autonomous delivery still faces substantial challenges, the capabilities of the technology are promising for a country like India, with a fragmented delivery system. There has also been conclusive evidence that autonomous delivery robots (ADRs) can bring carbon emissions down compared to traditional van deliveries, especially when the delivery areas are near to the depot [90]. The existing policy framework in India, however, does not allow testing of autonomous technology and significant research is required to assess the implementation challenges in enabling CATs, ETs, and ADRs in India. Recent policy initiatives by the Indian government, such as ‘Faster Adoption and Manufacturing of Hybrid and Electric Vehicles’ (FAME), are a valuable step towards fostering technology advancements in freight transport.
- **Crowdshipping on transit:** Crowdshipping on transit (COT) is a concept that incorporates the underutilized passenger transport mode capacity and related infrastructure to cover the last mile and deliver freight packages [91]. Packages are delivered with the help of commuters and other trip makers, who drop the packages off at designated places on their way, for the packages to then be picked up by another trip maker and delivered to the customer at the final destination. While large-scale formal crowdshipping programmes have been missing in Indian cities, the ongoing COVID-19 pandemic has put a sudden spotlight on introducing COT for enhancing non-ticket revenue of transit systems [92]. For instance, the public transit agency in Kerala, a Southern state in India, has recently initiated COT programs for parcel service in an attempt to overcome the fall in revenue following the pandemic-induced lockdowns and heightened risk perceptions [93]. Further research is required to scale up COT programs with the required infrastructure and operational efficiency for last-mile delivery.
- **Planning and cooperation initiatives:** Business establishments can achieve higher logistic performance by cooperating with other stakeholders in the freight system, utilizing resources more efficiently [94]. This cooperation can either be horizontal (between same types of establishments active in the same stage of supply chain, such as carriers) or vertical (between different establishments positioned upstream and downstream of a supply chain, such as shippers and carriers). Despite the potential benefits of collaboration among logistics service providers, there is little effective collaboration in practice. In the era of the sharing economy, logistics collaborations have great potential in a country like India towards on-demand logistics, freight consolidation, facility sharing, and warehousing.

## Conclusions

This paper has reviewed Indian freight transport research and policies in terms of logistic performance and solution approach for mitigating externalities. What emerges from the discussion is that freight transport is growing substantially in India, mainly due to its growing economy. However, like in Europe and the USA, the share of road transport seems too high, especially bearing in mind the higher negative externalities that road freight causes relative to its main competitor, rail freight. Decreasing this share is not easy because of the fragmentation of receivers and carriers in India. The penetration of the Internet has also triggered, like in most developed and many developing countries, an increase in B2CF, which only increases air pollutant and GHG emissions, and congestion. To make matters worse, some of the policies intended to reduce the externalities from road freight in India are proving counterproductive. Restricting areas or hours of freight deliveries, or banning big trucks, has increased, rather than decreased some externalities. The response has often been to fragment deliveries even further by, for example, using smaller vehicles and making more trips, or delivering during the night when time restrictions do not apply.

A portfolio of solution concepts to overcome the inefficiencies has also been presented. Although there is no one solution that will solve all the problems discussed, the following policy interventions have the potential to make freight more efficient in India and reduce emissions of air pollutants and GHG. Long-term planning and significant investment in infrastructure, including parking and loading bays, exclusive truck routes, consolidation centres, urban logistics spaces and pack stations, increasing road and rail network quality and capacity, could go some way towards integrating road and rail freight and reducing traffic congestion. Short-term planning to reduce the impact of freight transport within the existing expanse of transport infrastructure, including the development of intermodal logistic parks in tandem with dedicated freight corridors, and the promotion of double-stack clearance (stack containers one above the other) of intermodal corridors, would also help to increase the efficiency of the freight transport system in India. Additional interventions could entail developing more accurate demand forecasting models, automating warehouse processes, deploying inventory data insights in distribution network design to deal with demand volatility, implementing just-in-time inventory systems and fostering lean ordering behaviour among establishments, achieving efficiency in truck routing and dispatching through real-time information, implementing intelligent transport systems (ITS), delivering space booking systems, and route planning systems, and promoting electric delivery trucks.

The coordination and implementation of these actions are likely to require financial and time resources, and to encounter some degree of stakeholder backlash. This review has outlined the progress in freight research related to India and provided a framework of solution concepts. India is in a position to leapfrog and make important advances in policy implementation and doing so will increase the efficiency of freight transport, with consequent positive impacts on the economy and the environment.

## Data Availability

The data that support the findings of this study are available from Prasanta Sahu (prasantsahu222@gmail.com) upon reasonable request.
